# Q Fever Endocarditis in Romania: The First Cases Confirmed by Direct Sequencing

**DOI:** 10.3390/ijms12129504

**Published:** 2011-12-20

**Authors:** Ani Ioana Cotar, Daniela Badescu, Mihaela Oprea, Sorin Dinu, Otilia Banu, Dan Dobreanu, Minodora Dobreanu, Adina Ionac, Mirela Flonta, Monica Straut

**Affiliations:** 1 National Institute for Research in Microbiology and Immunology, Cantacuzino, Spl. Independentei 103, 050096, Bucharest, Romania; E-Mails: badescu.daniela@gmail.com (D.B.); moprea@cantacuzino.ro (M.O.); sorind@cantacuzino.ro (S.D.); mstraut@cantacuzino.ro (M.S.); 2 Institute for Emergency Cardiovascular Diseases Prof. C.C. Iliescu, Sos. Fundeni 258, 022328, Bucharest, Romania; E-Mail: otiliabanu@gmail.com; 3 Institute for Cardiovascular Diseases and Transplant Targu Mures, Str. Gheorghe Marinescu 50, 540136, Targu Mures, Mures, Romania; E-Mail: dobreanu@yahoo.com; 4 University of Medicine and Pharmacy Targu Mures, Str. Gheorghe Marinescu 38, 540139, Targu Mures, Mures, Romania; E-Mail: dobreanum@yahoo.com; 5 Institute for Cardiovascular Diseases Timisoara, Str. Gheorghe Adam, 13A, 300310, Timişoara, Timis, Romania; E-Mail: adina_ionac@rdstm.ro; 6 Clinical Hospital for Infectious Diseases Cluj-Napoca, Str. Iuliu Moldovan 23, 400348, Cluj-Napoca, Cluj, Romania; E-Mail: labinfecluj@yahoo.com

**Keywords:** chronic Q fever, blood culture-negative endocarditis, molecular diagnosis

## Abstract

Infective endocarditis (IE) is a serious, life-threatening disease with highly variable clinical signs, making its diagnostic a real challenge. A diagnosis is readily made if blood cultures are positive, but in 2.5 to 31% of all infective endocarditis cases, routine blood cultures are negative. In such situations, alternative diagnostic approaches are necessary. *Coxiella burnetii* and *Bartonella* spp. are the etiological agents of blood culture-negative endocarditis (BCNE) most frequently identified by serology. The purpose of this study is to investigate the usefulness of molecular assays, as complementary methods to the conventional serologic methods for the rapid confirmatory diagnostic of Q fever endocarditis in patients with BCNE. Currently, detection of *C. burnetii* by culture or an antiphase I IgG antibody titers >800 represents a major Duke criterion for defining IE, while a titers of >800 for IgG antibodies to either *B. henselae* or *B. quintana* is used for the diagnosis of endocarditis due to *Bartonella* spp. We used indirect immunofluorescence assays for the detection of IgG titers for *C. burnetii*, *B. henselae* and *B. quintana* in 57 serum samples from patients with clinical suspicion of IE. Thirty three samples originated from BCNE patients, whereas 24 were tested before obtaining the blood cultures results, which finally were positive. The results of serologic testing showed that nine out of 33 BCNE cases exhibited antiphase I *C. burnetii* IgG antibody titer >800, whereas none has IgG for *B. henselae* or *B. quintana*. Subsequently, we used nested-PCR assay for the amplification of *C. burnetii* DNA in the nine positive serum samples, and we obtained positive PCR results for all analyzed cases. Afterwards we used the DNA sequencing of amplicons for the repetitive element associated to *htpAB* gene to confirm the results of nested-PCR. The results of sequencing allowed us to confirm that *C. burnetii* is the causative microorganism responsible for BCNE. In conclusion, the nested PCR amplification followed by direct sequencing is a reliable and accurate method when applied to serum samples, and it may be used as an additional test to the serological methods for the confirmatory diagnosis of BCNE cases determined by *C. burnetii*.

## 1. Introduction

Infective endocarditis is a serious, life-threatening disease with highly variable clinical signs that are making the condition a diagnostic challenge. A diagnosis is readily made if blood cultures are positive, but in 2.5 to 31% of all infective endocarditis cases, routine blood cultures are negative [1–3]. This variation in incidence could be explained by several factors, including: (i) differences in the diagnostic criteria used; (ii) specific epidemiological factors, as for fastidious zoonotic agents; (iii) variations in the early use of antibiotics before the blood sampling; (iv) differences in sampling strategies; or (v) involvement of unknown pathogens [4,5].

Blood culture negative endocarditis (BCNE) was recognized by Osler at the beginning of last century [6,7]. Recently, many publications in European countries have demonstrated a significant involvement of *Coxiella burnetti*, *Bartonella henselae*, and *B. quintana* in patients with BCNE [8,9]. *C. burnetii* is one of the most encountered fastidious agents in BCNE. Q fever is characterized by its clinical polymorphism and the presentation of the disease is variable, with both acute and chronic manifestations [10,11]. Following acute infection, 1 to 5% of patients progress to chronic infection, which can develop after months to several years after acute Q fever infection, the longest interval being 20 years after infection [12,13].

Endocarditis is the main form of chronic Q fever (78% of all chronic Q fever cases) [14]. The most exposed persons are patients with preexistent valvular disease or vascular defects, especially aortic aneurysm and aortic stents and prostheses, immunocompromised patients, and pregnant women [15–18]. The estimated risk of transformation from acute infection to Q fever endocarditis in patients with preexisting valvulopathy is approximately 40% [17]. Because symptoms of Q fever endocarditis are protean and not specific, diagnosis is often delayed, only after significant valvular damage has occurred, resulting in an increasing mortality rate. Some authors proposed that all patients with acute Q fever be investigated by a transthoracic echocardiography [19].

The diagnosis of Q fever endocarditis requires both clinical endocarditis and isolation or serologic evidence of *C burnetii*. Because Q fever endocarditis is a chronic illness, a single serum specimen is sufficient for diagnosis. A phase I IgG titers of 800 or greater is one of the major modified Duke criteria [20]. Previously studies showed that PCR with serum samples may be helpful in establishing an early diagnosis of chronic Q fever [21].

Currently there is no data concerning the incidence of Q fever endocarditis cases among Romanian population. The first Q fever cases in Romania were registered in 1947 in Constanta County [22]. The most recent data about Q fever in Romania were represented by urban sporadic cases reported in the period 1981–1987 [23].

## 2. Results and Discussion

### 2.1. Case Definitions

Patients were considered to have definite blood culture-negative endocarditis if the results of standard blood cultures were negative, and if clinical and echocardiographic findings met the Duke criteria for infective endocarditis. All serum samples tested by serological methods for detection of IgG to *C. burnetii*, *B. quintana* and *B. henselae* originated from patients with clinical suspicion of BCNE. According to the modified Duke criteria a single positive blood culture for *Coxiella burnetii* or antiphase I IgG antibody titers >800 represents a major criterion for definite infective endocarditis [24]. The results of serological testing showed that 9 out of 33 serum samples exhibited antiphase I *C. burnetii* IgG antibody titers >800, while none of samples has IgG for *B. henselae* or *B. quintana*. The modified Duke criteria used for definite infective endocarditis (IE) diagnosis of analyzed patients with clinical suspicions of BCNE are presented in Table 1.

Diagnosis of IE is definite if there are 2 major criteria or 1 major and 3 minor criteria or 5 minor criteria [25,26]. Eight out of nine investigated cases fulfilled 2 major criteria (antiphase I *C. burnetii* IgG antibody titer >800 and when there is a vegetation or a new valvular regurgitation) for defining IE, while the remaining case fulfilled one major criterion (antiphase I *C. burnetii* IgG antibody titers >800) and one minor criterion (fever ≥ 38 °C), being classified as a possible case.

It is well-known that endocarditis is the most common presentation of chronic Q fever. Initially thought to be a rare disorder, later it has been estimated to account for up to 5% of all endocarditis cases worldwide [27]. It occurs almost exclusively in patients who have pre-existing valvular disease or who are immunocompromised. Unlike typical cases of endocarditis, the clinical presentation of endocarditis from chronic Q fever is often nonspecific and lacks many of the typical features of subacute, bacterial endocarditis, such as usual clinical and echocardiographic features common to typical cases of endocarditis [28]. Thus, the diagnosis is often significantly delayed or even missed, resulting in significant morbidity and mortality. Despite increasing awareness, recent studies show a mean delay of seven months from symptom onset to diagnosis [29,30]. Without prompt recognition and adequate antimicrobial therapy, the course of Q fever endocarditis is severe and potentially fatal [30].

The diagnosis of Q fever endocarditis is hampered by the inability to culture *C. burnetii* using routine media. As a strict obligate intracellular bacterium, it can only be cultured in living cell lines, or embryonated chicken eggs, but the cultures cannot be easily performed in most laboratories, and the technique is restricted to biosafety level 3 laboratories. Thus, the diagnosis of chronic Q fever, therefore, relies on serological testing, being characterized by increased titres against the phase I antigen.

### 2.2. PCR and Sequencing Results

Detection of *C. burnetii* DNA by PCR is an important diagnostic method that could be used on different types of clinical specimens (blood, serum, infected heart valves) [31]. During the last years, several PCR based diagnostic assays have been developed to detect *C. burnetii* DNA in cell cultures and clinical samples. These assays used conventional PCR [32], nested PCR [33–36] or real-time PCR conditions with LightCycler [37,38], SYBR Green [39] or TaqMan chemistry. The target sequences of the assays originated from single chromosomal genes like *com1*, on plasmids (QpH1, QpRS) or within the transposase gene of insertion element *IS1111* that is present in 20 copies in the genome of the *C. burnetii* Nine Mile RSA493 strain [40]. Due to the multicopy number of the *IS1111* element, the corresponding PCR is very sensitive.

**Figure 1:**
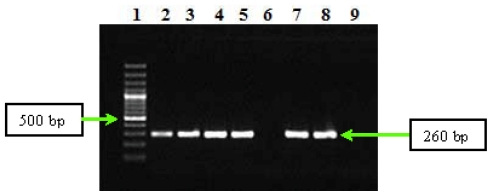
Gel electrophoresis of amplification products from the second round of repetitive element associated to *htpAB* gene in analyzed DNA samples of Q fever endocarditis cases. Line **1**—Gene Ruler 100 bp Plus DNA Ladder (Fermentas); **2**–**5** and **7**–**8**—DNA samples from Q fever endocarditis cases, **6**—negative serum for *C. burnetii*; **9**—negative control (pure water).

The results of PCR assays performed on DNA from serum samples positive for antiphase I *C. burnetii* IgG showed that nested-PCR assay permitted the amplification of *C. burnetii* DNA. Thus, nested-PCR led to obtaining the amplification products of the repetitive element *IS1111a* associated to *htpAB* transposase gene in the second round of amplification for all analyzed DNAs (Figure 1).

## 3. Experimental Section

### 3.1. Patients

In this study we analyzed the role of *C. burnetii* as causative agent of blood culture negative infective endocarditis among patients with clinical diagnostic of infective endocarditis. The blood-cultures were performed for 102 patients hospitalized in three Institutes for Cardiovascular Diseases from Bucharest, Timisoara and Targu-Mures, and in Clinical Hospital for Infectious Diseases from Cluj. For each patient, a standardized questionnaire was filled by the physician in charge and logged into a database. The information filled in the questionnaire were consisted of: known preexisting valvular defect, type of valve involved (native/bioprosthetic/mechanical valve, and its position: aortic, mitral, tricuspid, pulmonary); previous antibiotic therapy; clinical symptoms; and laboratory results. Patients were considered to have possible or definite endocarditis according to the modified Duke criteria.

### 3.2. Indirect Immunofluorescence Assays

57 serum samples were tested by indirect immunofluorescence assay (IFA) for detection of IgG antibodies to *C. burnetii*, *B. henselae* and *B. quintana*. From these samples 33 originated from patients with BCNE, and the rest of samples were tested before obtaining the blood cultures results, which finally were positive. We used IFA kits (Vircell, Spain) for detection of IgG antiphase I and antiphase II *C. burnetii*, and for IgG to *B. quintana* and IgG to *B. henseale.* The presence of IgG titers *>*800 to *C. burnetii* or *B. quintana or B. henselae* were considered positive for endocarditis diagnosis. Furthermore, we analyzed the positive serum samples for *C. burnetii* with antiphase I IgG antibody titers >800 using molecular methods for the confirmation of serological results.

### 3.3. Molecular Methods

#### 3.3.1. DNA Extraction

The serum samples from nine patients with antiphase I *C. burnetii* IgG antibody titer >800, were used for DNA extraction. Total genomic DNA was extracted from 200 microliters of serum using the QIAamp blood kit (Qiagen, Hilden, Germany) according to the manufacturer instructions. DNA was resuspended in fifty microliters of elution buffer. Genomic DNAs were stored at 4 °C until their use as templates in PCR assays and subsequently at −20 °C. DNA samples have been handled carefully to avoid the risk of cross-contamination. DNA extraction, mix preparation, and PCR were performed in different rooms to prevent PCR carryover contamination. No positive control was used to prevent lateral contamination (*i.e.*, contamination caused by PCR products amplified in other tubes in the same assay). DNA extracted from serum specimens of blood donors was used every 4 specimens as a negative control.

#### 3.3.2. PCR Assay

We used a nested-PCR assay for detection of the repetitive element *IS1111* associated to *htpAB* transposase gene (GenBank accession number M80806). This repetitive element is present in multiple copies in the genome of *C. burnetii* strains (e.g., there are 20 copies of this element in the *C. burnetii* Nine Mile I genome), which increase the detection sensitivity of this pathogen in serum samples [21]. In the first round of amplification we used IS111F1 and IS111R1 primers, which were designed to amplify a 485-bp fragment of the repetitive element *IS1111*, while the second round of amplification was performed using the IS111F2 and IS111R2 primers, which amplify an internal 260-bp fragment from the same target [27]. The sequence of specific primers used in nested-PCR reactions, and the molecular size of the amplicons are presented in Table 2.

In the nested-PCR assay, each gene fragment amplified separately on 2700 Applied Biosystems instrument using necessary components provided by Promega. The components used in each type of PCR reaction are described in Table 3. The parameters for the amplification cycles used in each PCR experiment are presented in Table 4. PCR products from nested-PCR assay were separated in a 1.5% agarose gel for 1 h at 100 V, stained with ethidium bromide and detected by UV transillumination.

#### 3.3.3. Sequencing of PCR Products and Sequence Analysis

The sequencing of the amplicons from nested-PCR has been used to confirm the PCR results. The amplicons from the second round of nested-PCR were sequenced in both directions using the BigDye V3.1 kit as described by the manufacturer. Sequencing products have been resolved using an ABI 3100 automated *Avant* Genetic Analyzer (Applied Biosystems). Sequence analysis was performed with BioEdit program, which permitted obtaining of the consensus sequences that were compared with similar sequences from BLAST. The sequences obtained showed a sequence similarity of 100% with that of the GenBank prototype strain sequence. Thus, sequencing of the amplicons from the second round of PCR reaction has permitted to confirm that amplification products belong to *C. burnetii*. These two molecular tests were used together for the first time to investigate the BCNE cases with *C. burnetii* in Romania.

## 4. Conclusions

We propose that all patients with clinical suspicion of IE be tested serologically for evidence of infection with other agents such as *C. burnetii* in parallel with performing blood cultures.

This is the first report in this country for using the molecular methods to confirm Q fever endocarditis cases on the serum samples from eight confirmed cases and from one possible case of Q fever endocarditis tested by nested-PCR, based on repetitive element *IS1111a* of the transposase gene. This assay exhibited high sensitivity and specificity and led us to obtain specific amplification products, which were subsequently confirmed by direct sequencing to belong to *C. burnetii*, confirming previous studies showing that this nested-PCR assay presents a specificity of 100% and a sensitivity of one *C. burnetii* DNA copy [27]. In conclusion, our results have demonstrated that nested-PCR amplification, followed by direct sequencing, is a reliable and accurate method when applied to serum samples and can be used as a supplementary diagnosis tool for BCNE cases.

## Figures and Tables

**Table 1:** The modified Duke criteria used for defining infective endocarditis (IE) diagnosis applied to the nine patients.

		Major criteria			
Case	Age (years)/sex	Antiphase I *C. burnetii* IgG antibody titer >800	Evidence of endocardial involvement— Echocardiography positive for IE-vegetation	Evidence of endocardial involvement—New valvular regurgitation	Minor criteria
1	51/M	Present	MV	MV; AV	Fever ≥ 38 °C
2	62/F	Present	-	MV	Fever ≥ 38 °C
3	58/M	Present	AV	AV	Fever ≥ 38 °C
4	64/M	Present	MV	AV; MV	Fever ≥ 38 °C
5	60/M	Present	AV; MV	AV; MV	Fever ≥ 38 °C
6	57/M	Present	-	-	Fever ≥ 38 °C
7	70/F	Present	MV	AV; MV	Fever ≥ 38 °C
8	64/M	Present	AV	AV	Fever ≥ 38 °C
9	60/M	Present	MV	AV; MV	Fever ≥ 38 °C

Note: MV—mitral valve; AV—aortic valve.

**Table 2:** The primer sequences used in nested-PCR assay for the repetitive element *IS1111a* of *htpAB* transposase.

The gene	Primer	Nucleotide sequence	Amplicon size (bp)
*IS1111*	IS111F1	5′-TACTGGGTGTTGATATTGC-3′	485
	IS111R1	5′-CCGTTTCATCCGCGGTG-3′	
	IS111F2	5′-GTAAAGTGATCTACACGA-3′	260
	IS111R2	5′-TTAACAGCGCTTGAACGT-3′	

**Table 3:** The components used in nested-PCR reactions.

The components used in nested-PCR						
Primers conc.	MgCl_2_ conc.	dNTP mix conc.	DNA TaqPol conc.	TaqPol buffer conc.	DNA conc.	Volume/reaction
0.3 μM	2.5 mM	0.2 mM	0.025 U/μL	1×	50 ng/μL	50 μL

**Table 4:** The conditions used for the amplification of repetitive element *IS1111* of *htpAB* transposase gene.

	The amplification program for nested PCR assay					
Gene	Initial denaturation	No. of cycles	Denaturation in each cycle	Annealing	Primers extension	Final extension
IS1111—first round	94 °C, 5 min	35	94 °C, 1 min	52 °C, 1 min	72 °C, 1 min	72 °C, 5 min
IS1111—second round				48 °C, 1 min		
